# Maternal and Zygotic *aldh1a2* Activity Is Required for Pancreas Development in Zebrafish

**DOI:** 10.1371/journal.pone.0008261

**Published:** 2009-12-11

**Authors:** Kristen Alexa, Seong-Kyu Choe, Nicolas Hirsch, Letitiah Etheridge, Elizabeth Laver, Charles G. Sagerström

**Affiliations:** Department of Biochemistry and Molecular Pharmacology, University of Massachusetts Medical School, Worcester, Massachusetts, United States of America; University of Texas MD Anderson Cancer Center, United States of America

## Abstract

We have isolated and characterized a novel zebrafish pancreas mutant. Mutant embryos lack expression of *isl1* and *sst* in the endocrine pancreas, but retain *isl1* expression in the CNS. Non-endocrine endodermal gene expression is less affected in the mutant, with varying degrees of residual expression observed for *pdx1*, *carbA*, *hhex*, *prox1*, *sid4*, *transferrin* and *ifabp*. In addition, mutant embryos display a swollen pericardium and lack fin buds. Genetic mapping revealed a mutation resulting in a glycine to arginine change in the catalytic domain of the *aldh1a2* gene, which is required for the production of retinoic acid from vitamin A. Comparison of our mutant (*aldh1a2^um22^*) to *neckless* (*aldh1a2^i26^*), a previously identified *aldh1a2* mutant, revealed similarities in residual endodermal gene expression. In contrast, treatment with DEAB (diethylaminobenzaldehyde), a competitive reversible inhibitor of Aldh enzymes, produces a more severe phenotype with complete loss of endodermal gene expression, indicating that a source of Aldh activity persists in both mutants. We find that mRNA from the *aldh1a2^um22^* mutant allele is inactive, indicating that it represents a null allele. Instead, the residual Aldh activity is likely due to maternal *aldh1a2*, since we find that translation-blocking, but not splice-blocking, *aldh1a2* morpholinos produce a phenotype similar to DEAB treatment. We conclude that Aldh1a2 is the primary Aldh acting during pancreas development and that maternal Aldh1a2 activity persists in *aldh1a2^um22^* and *aldh1a2^i26^* mutant embryos.

## Introduction

Similar to the pancreas of other vertebrates, the zebrafish pancreas consists of an endocrine and an exocrine portion. The zebrafish exocrine pancreas consists of acinar cells that release digestive enzymes into the intestine and the endocrine pancreas is composed of five cell types that secrete hormones directly into the blood stream; insulin producing β-cells, somatostatin producing δ-cells, glucagon producing α-cells, pancreatic polypeptide hormone secreting PP-cells and ghrelin producing ε-cells [1], [2]. The zebrafish pancreas develops from a dorsal and a ventral bud associated with the gut tube, where the dorsal bud is located slightly posterior to the ventral bud [3], [4]. The dorsal bud is the first to form at 24 hpf and eventually gives rise to endocrine pancreas. By 40 hpf, the ventral bud has formed and is composed of exocrine cells as well as a few endocrine cells. By 52 hpf, the two buds have merged to form one organ on the right side of the embryo, consisting of a single islet of endocrine cells surrounded by the exocrine pancreas [3], [4].

As in other vertebrates, expression of *pdx1* marks the future position of the pancreas in zebrafish embryos [2], [5], [6]. Zebrafish *pdx1* expression is first observed at 14 hpf [7], [8]; but cell transplantations have demonstrated endoderm commitment as early as 5 hpf [9]. At this early point, endoderm cells express *sox17*, a gene necessary for endoderm development [9], [10], [11]. Various intercellular signaling molecules act on these early endodermal cells to direct their differentiation into organs such as the pancreas. These factors include sonic hedgehog (Shh), bone morphogenetic protein (Bmp), transforming growth factor β (TGF-β), fibroblast growth factor (Fgf) and retinoic acid (RA) [6], [7], [12], [13], [14], [15], [16].

RA is involved in the formation of the central nervous system, lung, kidney, intestine, and pancreas [12], [15], [17], [18], [19], [20]. In particular, RA is needed at the end of gastrulation for pancreas development and blocking RA signaling in zebrafish embryos prevents pancreas formation [15]. Accordingly, exogenously applied RA induces ectopic pancreatic gene expression in the anterior endoderm [15]. Experiments in amphibian and avian models give similar results, indicating a vertebrate requirement for RA in pancreas development [7], [12], [13]. RA is a small lipophilic molecule derived from dietary vitamin A (retinol). Retinol is converted to an aldehyde (retinaldehyde) which is further converted to a carboxylic acid (retinoic acid). The first step, oxidation of retinol to retinaldehyde, is made possible by several retinol dehydrogenases (RDHs) that have widespread and overlapping expression patterns. The second step, oxidation of retinaldehyde to RA, is carried out by retinaldehyde dehydrogenases (Raldh or Aldh), which have more tissue specific expression patterns [21], [22], [23], [24], [25]. In particular, *aldh1a2* (*raldh2*) is the major retinoic acid generating enzyme in the early mouse embryo and was thought until recently to be the only *raldh* expressed in zebrafish. Recently, *aldh1a3 (raldh3)* and *aldh8a1 (raldh4)* were identified in zebrafish [26], [27] but *aldh1a1 (raldh1)* has not been found in zebrafish to date. *aldh1a3* is expressed in the developing eye and ear after gastrulation and *aldh8a1* is expressed later around 2 dpf in the liver and intestine [26], [27] suggesting that these genes are not involved in early pancreas development. In contrast, *aldh1a2* is expressed at 30% epiboly in the mesendoderm and continues to be expressed in the posterior and lateral mesoderm during segmentation [28]. At later stages, *aldh1a2* is expressed in the somites and the pronephric anlage (by 15 hpf) as well as in pharyngeal arch and pectoral fin mesenchyme (32 hpf) [28], [29], [30], [31], [32], [33]. Expression of *aldh1a2* adjacent to, but not within, the pancreatic anlage is consistent with observations that the anterior paraxial mesoderm is a source of RA driving pancreas formation. Accordingly, three Retinoic Acid Receptors (two RARα and one RARγ) are expressed in the endoderm, indicating that the RA signal can be received directly in the endoderm [8].

We carried out a haploid ENU (N-ethyl-N-nitrosourea) screen for endocrine pancreas mutations and discovered a mutant (88.21) that does not develop *isl1* expression in the endocrine pancreas, but maintains *isl1* expression in the CNS. More detailed analysis of the 88.21 mutant revealed residual expression of several pancreas (e.g. *pdx1*) and liver (e.g. *hhex* and *prox1*) genes, suggesting that endoderm organ differentiation, including pancreas formation, is not completely lost in the mutant. We mapped the 88.21 mutant using a CA panel and identified a mutation in the catalytic domain of the *aldh1a2* gene; therefore we named our mutant *aldh1a2^um22^*. Two other mutant alleles for *aldh1a2* have been reported, *neckless* (*nls* or *aldh1a2^i26/i26^*; a point mutation in the NAD binding domain) and *no fin* (*nof* or *aldh1a2^u11/u11^*; a point mutation in the catalytic domain) [28], [34]. A detailed analysis of endoderm gene expression in *aldh1a2^i26^* embryos revealed residual expression of several endoderm markers, (e.g. *pdx1*), similar to the phenotype seen in *aldh1a2^um22^* mutants. In contrast, we find that embryos treated with DEAB (diethylaminobenzaldehyde), a competitive reversible inhibitor of all Aldhs, completely lack expression of all pancreas and liver genes, indicating that there is residual Aldh activity in *aldh1a2^um22^* and *aldh1a2^i26^* mutant embryos. Notably, targeting both maternal and zygotic transcripts using MOs to the *aldh1a2* translation start site produces a phenotype comparable to DEAB treatment. In contrast, targeting primarily zygotic transcripts using MOs to the exon1/intron1 splice site of *aldh1a2* does not fully block endodermal gene expression. Our results reveal an absolute requirement for Aldh activity in pancreas development and demonstrate residual Aldh activity in *aldh1a2^um22^* and *aldh1a2^i26^* mutants, likely due to maternally contributed Aldh1a2.

## Materials and Methods

### Fish Maintenance

Ekkwill (EK), Tupfel long fin (TL) and *neckless (aldh1a2^i26^)* (Gift from Prince Lab) embryo*s* were collected from natural matings and reared in 1/3 Ringer's. Embryos were staged using morphological criteria up to 24 hours post fertilization (hpf) and then by time of development at 28.5°C [35].

### ENU Screen

EK males were treated with 3 mM ENU (N-ethyl-N-nitrosourea) once a week for 3 weeks. The males were then crossed repeatedly to clean out any post meiotic germ cells that were mutagenized. Mutagenized males were then crossed to EK females and the progeny (F1) were raised. Haploid embryos were produced by In Vitro Fertilization (IVF) of F1 female progeny with irradiated sperm. Haploid embryos were raised to approximately 30 hpf and fixed in 4% paraformaldehyde for in situ hybridization with *islet1 (isl1)* probe. Embryos were screened based on *isl1* expression. F1 females that produced embryos with mutant phenotypes were out-crossed to TL males and the progeny (F2) were raised and in-crossed for recovery of mutation in diploid embryos.

### Mapping, DNA Extraction, RNA Extraction and cDNA Synthesis

Mutant carriers were in-crossed and progeny raised to 4 dpf. Embryos were sorted based on their phenotype; mutants develop a swollen pericardium and lack fin buds. Genomic DNA was extracted from phenotypically mutant and phenotypically wild type embryos at day 4. DNA pools were created from phenotypically mutant and wild type embryos. Bulk segregant analysis was performed on the DNA pools using a 192 CA marker panel [36], [37], [38]. Two markers were found to be linked to the mutation: z10441 (FW:GCATTCAGATTCTGGGGTGT, RV: CGGATGAACCCATCAATCTC) and z8693 (FW: GCTTTTTGAGCAGATGAGGC, RV: CATGTACGCGTTGACTTTGC). PCR was performed on individual embryos using the same primers. cDNA was synthesized from RNA extracted from pools of 10 phenotypically mutant and 10 phenotypically wild type embryos using Invitrogen Superscript III Reverse Transcriptase Kit. PCR primers, FW: CCAAAGTTGTAATCGCACATC, RV:TTTTTTTTTTTTTTTCAGAGGTAAAAC, were used to clone full-length *aldh1a2* cDNA. Stratagene Hi Fi taq polymerase was used in the PCR and the product was sequenced. Primers FW: AGCGGCCGTCTTCCCAGAGATATC and RV: GGAATGGGTGTAGGCAGTTAATGGTGG were used to sequence *aldh1a2* from individual embryos.

### mRNA and Morpholino Injections

An antisense morpholino oligo (MO) designed to block translation of the *aldh1a2* mRNA (tMO) 5′GCAGTTCAACTTCACTGGAGGTCAT3′ [28] and one control mismatch morpholino (mmMO 5′GCAcTTgAACTTCAgTGGAcGTgAT3′ that has five mismatches relative to tMO) were obtained from Gene Tools. 1 nl of 100 uM, 250 uM, 500 uM, 750 uM and 950 uM of tMO was injected at the 1–2 cell stage. A splice MO (sMO) designed to exon1/intron1 splice junctions: 5′TTGAAAAAGTCCGACAAACCTTGGT3′ and one control morpholino (mmMO: 5′TTcAAAAAcTCgGACAAtCCTTcGT3′ with five mismatches relative to sMO) was obtained from Gene tools. 1 nl of 500 uM, 750 uM, and 950 uM of sMO was injected at the 1–2 cell stage.

For rescue experiments, the *aldh1a2* ORF was amplified from TL embryos or *aldh1a2^um22^* mutant embryos using FW: ATGACCTCCAGTGAAGTTGAACTGCCA and RV:TTAAGACGTCTTGCTTCATCGTAATGGTTTTCA. Both ORFs were cloned with Invitrogen Topo TA Cloning kit, digested using EcoR1 and cloned into PCS2+. Constructs were linearized with Not1 and Ambion Kit Sp6 was used to make mRNA. 500 pg of mRNA was injected into an *aldh1a2^um22^* in-cross at 1–2 cell stage. mRNA and MO injected embryos were fixed in 4% paraformaldehyde at various developmental stages for in situ hybridization.

### DEAB Treatment

A 1 mM stock of DEAB was dissolved in DMSO. Embryos were treated in the dark with 1 uM, 5 uM and 10 uM of DEAB dissolved in 1X PTU at 6, 8, 10, or 12 hpf. Embryos were fixed at various stages and assayed by in situ hybridization. Control embryos were treated in DMSO under similar conditions.

### In Situ Hybridization

Antisense digoxigenin- and fluorescein-labeled probes were produced by standard methods. The *krx20*, *myosin heavy chain* (*mhc*), *insulin*, *sid4*, *carbA*, *pdx1*, *isl1*, *transferrin*, *p48*, *somatostatin*, *ifabp (intestinal fatty acid binding protein)* and *shh* probes used were described previously [39], [40], [41]. Full-length *prox1* was obtained from Open Biosystems, One- and two-color in situ hybridization was carried out as described previously [39], [40].

### RT-PCR

RT-PCR was performed using a Qiagen PCR Kit (Cat. No 204054) and cDNA synthesized from wild type embryos at 3 and 6 hpf. RNA was extracted from 10 wild type embryos at 3 hpf or 6 hpf and cDNA was synthesized using Invitrogen Superscript III Reverse Transcriptase Kit. The following primers were used to obtain PCR product: BActin FW: ATACACAGCCATGGATGAGGAATTCC and RV: GGTCGTCCAACAATGGAGGGGAAAA, Tubulin 1 FW: AAGAGATGACGCAGTCTGTCGTAGTC and RV: AGAAGCTCGTCAGCGCGTCATCATAA, Odc-1 FW: TTTGACTTCGCCTTCCTGGAGGAGGG and RV: CCCCAGATCCGCCACATAGAAGGCAT, *aldh1a2^1-2^* FW: ATGACCTCCAGTGAAGTTGAACTGC and RV: CTTGTCGGATTCCTGGACATCACAG, and *aldh1a2^10-11^* FW: GCAAAGCTCCTCCTACTAAAGGCTTCTTC and RV: TTCTGTGTTGTTGGCTCTCTCAATCACT.

## Results

### An ENU Screen for Zebrafish Pancreas Mutants

A haploid in situ hybridization screen of ENU (N-ethyl-N-nitrosourea) mutagenized zebrafish was carried out to identify mutations in endocrine pancreas development. Ekkwill (EK) males were treated with 3 mM ENU and crossed to EK females. F1 progeny was raised and eggs from F1 females were in vitro fertilized using irradiated sperm from EK males. The resulting haploid embryos were raised until 30 hpf and assayed by in situ hybridization for *islet1* (*isl1*) expression to detect defects in the endocrine pancreas. F1 females that produced clutches with 50% mutant embryos were outcrossed to Tupfel long fin (TL) males. F2 progeny were raised and screened for recovery of the mutation in the F3 generation. We screened 200 genomes and discovered ten females with defective endocrine pancreas formation. Six of the ten females died, developed tumors or did not produce progeny. Out of the remaining four females, we recovered diploid mutants for two.

**Figure 1:**
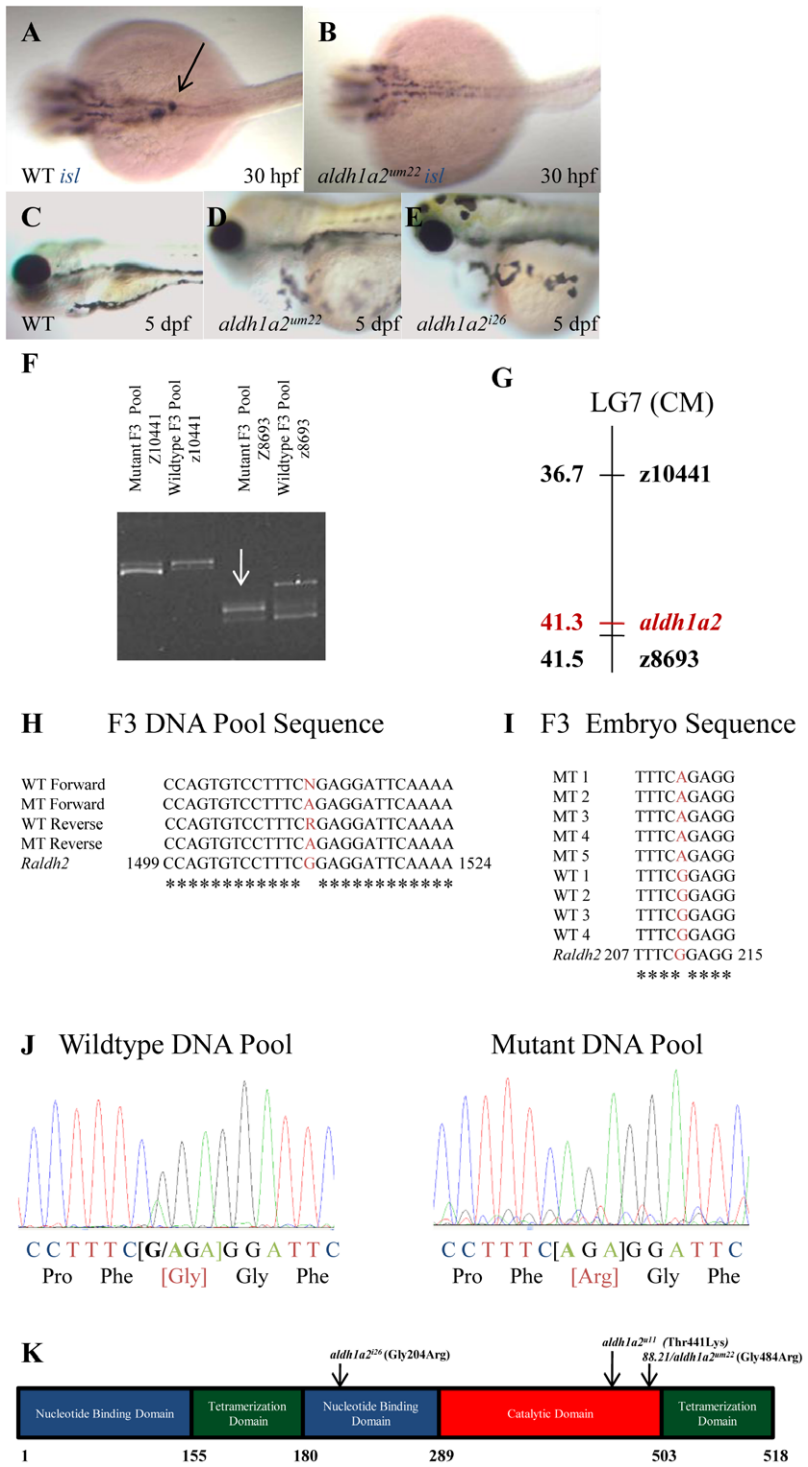
88.21 is a novel *aldh1a2* allele. A, B. *Islet1* (*isl1*) expression was used in a haploid ENU screen to identify mutants in endocrine pancreas development. Dorsal view of 30 hpf wild type embryo with *isl1* expression in the CNS and endocrine pancreas (A; black arrow indicates expression in pancreas) and 88.21 mutant embryo with *isl1* expression in the CNS, but not in the endoderm (B). C–E. Lateral view of live wild type (C), 88.21 (D), and *neckless aldh1a2^i26^* (E) embryos at day 5. F. Linkage analysis using CA repeat markers on pooled genomic DNA from 88.21 mutants and pooled genomic wild type DNA. Marker z10441 amplifies a 450 bp band and a faint 500 bp band in the mutant pool compared to a faint 450 bp band and a 500 bp band in the wild type pool. Marker z8693 amplifies two bands at 250 bp and 300 bp in the mutant pool compared to 250 bp, 300 bp as well as a 400 bp band in the wild type pool. White arrow points to lack of 400 bp band in mutant. G. Schematic drawing of part of linkage group 7 (LG7), showing the location of z10441 and z8693 and *aldh1a2* (in red) in reference to these markers. H–J. Sequence analysis of pooled 88.21 mutant (MT) genomic DNA and pooled wild type (WT) genomic DNA (H, J), as well as of individual mutant (MT) and wild type (WT) embryos (I). 88.21 fish carry a mutation that converts Gly^484^ to Arg (in red, and outlined in brackets in J) located in the catalytic domain. K. Schematic of Aldh1a2 protein and the location of the *aldh1a2* mutant alleles *aldh1a2^i26^*, *aldh1a2^u11^* and *88.21/aldh1a2^um22^*.

Embryos from one of the recovered mutants (88.21) lack *isl1* expression in the endocrine pancreas, but maintain expression in the CNS (Figure 1B versus wild type in Figure 1A). 88.21 embryos first display a morphological phenotype approximately at day 4, as they do not develop fin buds and have a swollen pericardium (Figure 1D versus wildype in Figure 1C). Since the EK and TL strains used in our screen are highly polymorphic with respect to their CA repeats, we used a PCR panel consisting of 192 primer pairs that amplify CA repeats in the zebrafish genome to map the position of the mutation [36], [37], [38]. Specifically, genomic DNA pools from phenotypically wild type and phenotypically mutant embryos were amplified using primers from the CA marker panel. Based on the bulk segregant analysis of the DNA pools, two markers, z10441 and z8693, were found to be linked to the mutation (Figure 1F). Subsequent PCR of inividual embryos (not shown) confirmed the linkage. We detected three crossovers out of 44 meioses for the z10441 marker, which places the mutation approximately 7 cM away from this marker on linkage group 7 (Figure 1G).

### The 88.21 Mutant Represents a Novel *aldh1a2* Allele

A closer examination revealed that the z10441 and z8693 markers are both located near the *aldh1a2* (*raldh2*) gene on chromosome 7. As noted, *aldh1a2* is a retinaldehyde dehydrogenase (Raldh) involved in RA synthesis and there are two previously reported *aldh1a2* mutants, *neckless* (*nls* or *aldh1a2^i26^*; Figure 1E) and *no fin* (*nof* or *aldh1a2^u11^*) [28], [34]. Since the 88.21 mutant phenotype bears some resemblance to the *aldh1a2^i26^* phenotype (Figure 1D, E) – lack of pectoral fins, swollen pericardium and embryonic lethality by day 6 - we tested if 88.21 might represent a novel *aldh1a2* allele. To this end, we amplified full length *aldh1a2* from cDNA prepared from mutant and wild type embryo pools derived from an 88.21 incross. Sequencing of the PCR products identified a G to A change in the mutant pool that converts a glycine to an arginine at position 484 (Figure 1H, J) in the catalytic domain of Aldh1a2 (Figure 1K). Sequencing cDNA from individual embryos confirmed this change (Figure 1I).

**Figure 2:**
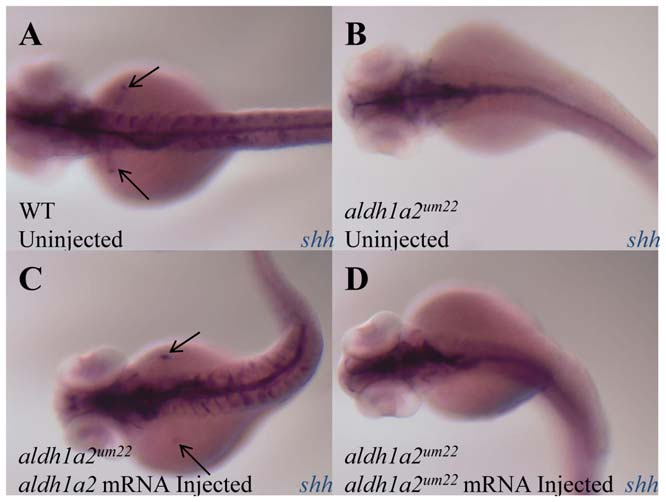
Wild type *aldh1a2* mRNA rescues 88.21 fin bud development. Dorsal views of 48 hpf embryos with *sonic hedgehog* (*shh*) expression in purple. A. Uninjected wild type embryo with *shh* expression in the CNS and fin buds (black arrows). B. *aldh1a2^um22^* mutant embryos lack *shh* expression in the fin buds. C. *aldh1a2^um22^* mutant embryo injected with *aldh1a2* wild type mRNA shows rescued fin bud expression (black arrows). D. *aldh1a2^um22^* mutant embryo injected with *aldh1a2^um22^* mutant mRNA is not rescued.

To confirm that the 88.21 phenotype is caused by a mutation in the *aldh1a2* gene, we set out to rescue the mutant phenotype with wild type *aldh1a2* mRNA. We find that 26% of embryos from an incross of 88.21 heterozygotes fail to develop fin buds (Table 1), as assayed by *shh* expression in fin buds at 48 hpf (Figure 2A, B) or by visual inspection for fin bud formation at 72 hpf (not shown). However, following injection of wild type *aldh1a2* mRNA at the 1–2 cell stage, only 8.5% of embryos lack fin buds, demonstrating that *aldh1a2* mRNA rescues fin bud development (Table 1, Fig. 2C). In contrast, injection of *aldh1a2* mRNA containing the 88.21 mutation does not rescue fin bud development (24% lack fin buds; Table 1, Fig. 2D). Notably, the swollen pericardium phenotype was not rescued by injection of *aldh1a2* mRNA. This result is consistent with previous work showing that fin bud development in *aldh1a2^i26^* and *aldh1a2^u11^* can be rescued by injecting wild type *aldh1a2* mRNA, but the swollen pericardium cannot be rescued [28], [34]. We conclude that the 88.21 mutation occurs in the *aldh1a2* catalytic domain and we refer to it as *aldh1a2^um22^*. Since the mutant mRNA appears to be inactive even when overexpressed, the *aldh1a2^um22^* allele is likely to represent a null allele. In particular, replacing a small conserved glycine residue with a large arginine in the catalytic domain may affect the function or folding of the Aldh1a2 protein.

**Table 1:** Rescue *88.21* fin bud development.

	Finbud	No Finbud
Uninjected	113/153 (74%)	40/153 (26%)
Injected aldh1a2 mRNA	107/117 (91.5%)	10/117 (8.5%)
Injected 88.21/aldh1a2^um22^ mRNA	61/80 (76%)	19/80 (24%)

Embryos from an incross of 88.21 heterozygotes were injected with 500 pg of wild type *aldh1a2* mRNA or *aldh1a2* mRNA containing the 88.21 mutation. Embryos were either fixed and assayed for fin bud expression by *shh* probe at 48 hpf or observed live at 72 hpf for fin bud development. Wild type *aldh1a2* rescued fin bud development (91.5% of embryos have fin buds) whereas *aldh1a2* carrying the 88.21 mutation did not rescue (76% of embryos have fin buds).

### Endoderm Gene Expression Is Variably Affected in *aldh1a2^um22^* and *aldh1a2^i26^* Mutants

**Figure 3:**
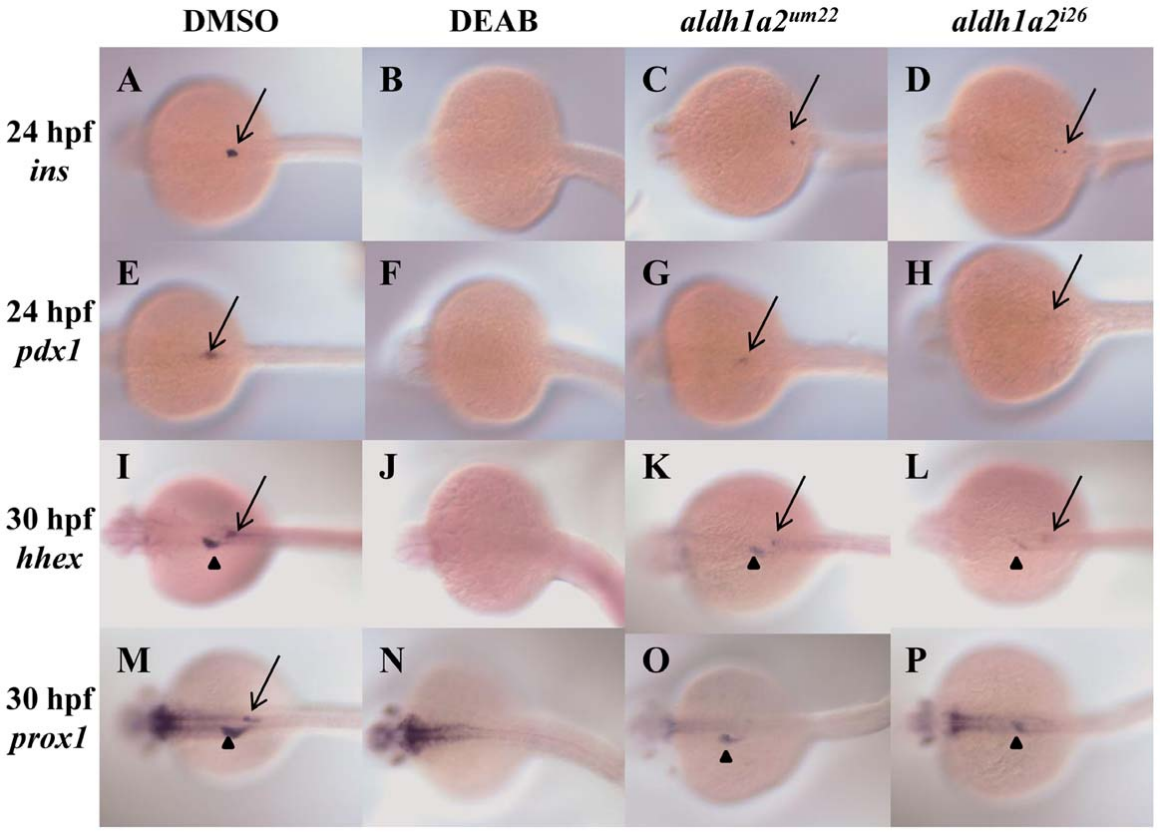
*aldh1a2^um22^* and *aldh1a2^i26^* mutant embryos retain some endoderm gene expression at 24 and 30 hpf. DMSO treated wild type embryos (A, E, I, M), DEAB-treated wild type embryos (B, F, J, N), embryos from an incross of *aldh1a2^um22^* heterozygotes (C, G, K, O) and embryos from an incross of *aldh1a2^i26^* heterozygotes (D, H, L, P) were assayed for expression of *ins* at 24 hpf (A–D; black arrows indicate residual expression), *pdx1* at 24 hpf (E–H; black arrows indicate residual expression), *hhex* at 30 hpf (I–L; residual expression is indicated in pancreas (arrow) and liver (arrowhead)) and *prox1* at 30 hpf (M–P; residual expression is indicated in liver (arrowhead)). Embryos are in dorsal view with anterior to the left. See Table 2 for quantification.

**Figure 4:**
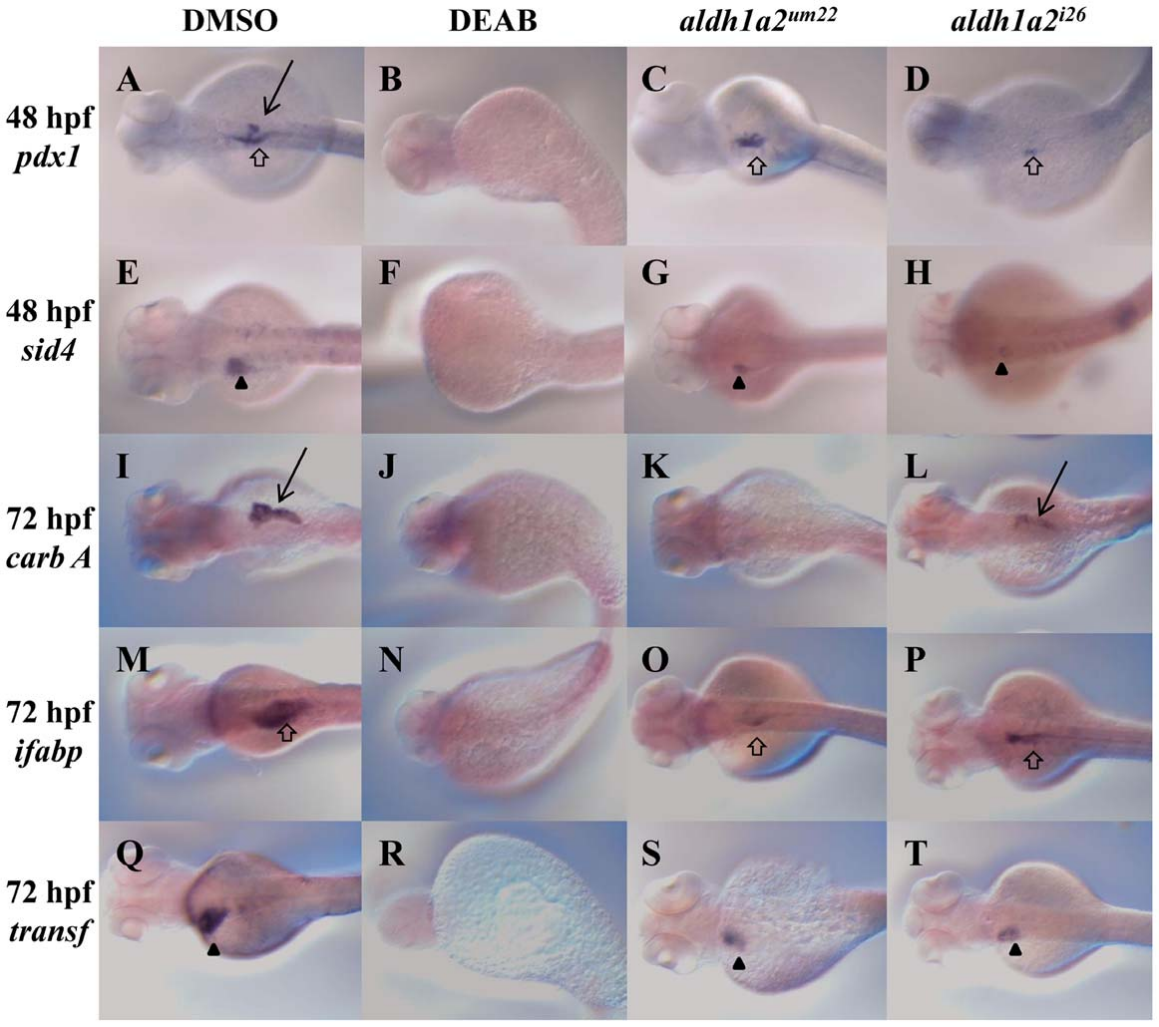
*aldh1a2^um22^* and *aldh1a2^i26^* mutant embryos retain some endoderm gene expression at 48 and 72 hpf. DMSO treated wild type embryos (A, E, I, M, Q), DEAB-treated wild type embryos (B, F, J, N, R), embryos from an incross of *aldh1a2^um22^* heterozygotes (C, G, K, O, S) and embryos from an incross of *aldh1a2^i26^* heterozygotes (D, H, L, P, T) were assayed for expression of *pdx1* at 48 hpf (A–D), *sid4* at 48 hpf (E–H), *carbA* at 72 hpf (I–L), *ifabp* at 72 hpf (M–P) and *transf* at 72 hpf (Q–T). Gene expression is observed in the intestine (open arrows), liver (black arrowheads) and pancreas (black arrows). Embryos are in dorsal view with anterior to the left. See Table 2 for quantification.

We observe variable effects on endoderm gene expression in *aldh1a2^um22^* mutants and we therefore compared the *aldh1a2^um22^* phenotype to the *aldh1a2^i26^* phenotype. The *aldh1a2^i26^* allele was previously analyzed with some endodermal markers [15] but we have expanded the analysis further. We find that endocrine-specific genes such as *isl1* (Table 2) and *sst1* (Table 2) are completely lost in both mutants at 24–30 hpf, as is *p48* expression in the exocrine pancreas (Table 2). In contrast, *pdx1* expression remains in the majority of both *aldh1a2^i26^* and *aldh1a2^um22^* mutant embryos (Figure 3E versus 3G, H; Figure 4A versus 4C, D; Table 2), as does *carboxypeptidase A (carbA)* expression, although *carbA* expression is more pronounced in *aldh1a2^i26^* (Figure 4I versus 4K, L; Table 2). We also find that expression of *hhex* and *prox1* (that are expressed in both the ventral pancreatic bud and the liver) persists in both mutants (Figure 3I versus 3K, L and 3M versus 3O, P; Table 2). Analyzing other liver markers later in development revealed that expression of both *sid4* (at 48 hpf) and *transferrin* (*transf*, at 72 hpf) persists in both *aldh1a2^um22^* and *aldh1a2^i26^* mutant embryos (Figure 4E versus 4G, H; 4Q versus 4S, T; Table 2). *intestinal fatty acid binding protein* (*ifabp*) expression is decreased at 72 hpf (Figure 4M versus 4O, P; Table 2) in *aldh1a2^um22^* and *aldh1a2^i26^* mutant embryos, suggesting that differentiation of the intestine takes place, although perhaps not to completion. Expression of the early endoderm marker *sox17* is maintained (data not shown). Also, while our data suggest that endocrine gene expression may be most sensitive to the loss of Aldh1a2 function, we find that *insulin* (*ins*) expression remains in some mutant embryos at 24, 48 and 72 hpf, suggesting that endocrine gene expression is not completely blocked in the mutants (Fig. 3C, D; Table 2). Lastly, we tested whether embryos with residual expression of one endoderm gene had residual expression of other endoderm genes, but did not observe a correlation, suggesting that expression of each gene varies from embryo to embryo (Figure S1).

**Table 2:** DEAB, *aldh1a2^um22^*, and *aldh1a2^i26^* in situ results.

Hpf^1^	Marker	DEAB			aldh1a2^um22^			aldh1a2^i26^		
		None^2^	Weak^2^	Wild type^2^	None^2^	Weak^2^	Wild type^2^	None^2^	Weak^2^	Wild type^2^
24	*ins*	100% (99/99)	0% (0/0)	0% (0/0)	22% (37/167)	7% (12/167)	71% (118/167)	16% (13/79)	13% (10/79)	71% (56/79)
24	*pdx1*	100% (40/40)	0% (0/0)	0% (0/0)	0% (0/115)	24% (28/115)	76% (87/115)	15% (16/105)	9% (9/105)	76% (80/105)
30	*hhex*	100% (88/88)	0% (0/0)	0% (0/0)	14% (32/228)	12% (28/228)	74% (168/228)	12% (14/116)	15% (17/116)	73% (85/116)
30	*isl1*	100% (26/26)	0% (0/0)	0% (0/0)	25% (26/104)	0% (0/104)	75% (78/104)	26% (13/50)	0% (0/50)	74% (37/50)
30	*p48*	100% (27/27)	0% (0/0)	0% (0/0)	26% (32/125)	0% (0/125)	74% (93/125)	25% (16/64)	0% (0/64)	75% (48/64)
30	*prox1*	100% (93/93)	0% (0/0)	0% (0/0)	0% (0/63)	24% (15/63)	76% (48/63)	0% (0/195)	26% (50/195)	74% (145/195)
30	*sst*	100% (23/23)	0% (0/0)	0% (0/0)	24% (22/92)	0% (0/92)	76% (70/92)	26% (21/80)	0% (0/80)	74% (59/80)
48	*ins*	100% (25/25)	0% (0/0)	0% (0/0)	16% (10/63)	8% (5/63)	76% (48/63)	15% (12/78)	10% (8/78)	74% (58/78)
48	*pdx1*	100% (95/95)	0% (0/0)	0% (0/0)	0% (0/167)	25% (41/167)	75% (126/167)	12% (9/76)	12% (9/76)	76% (58/76)
48	*shh*	100% (24/24)	0% (0/0)	0% (0/0)	24% (13/54)	0% (0/54)	76% (41/54)	24% (15/62)	0% (0/62)	76% (47/62)
48	*sid4*	100% (87/87)	0% (0/0)	0% (0/0)	6% (12/189)	20% (38/189)	74% (139/189)	12% (18/146)	12% (18/146)	75% (110/146)
72	*carbA*	100% (33/33)	0% (0/0)	0% (0/0)	23% (57/245)	1% (2/245)	76% (186/245)	11% (13/117)	15% (18/117)	74% (86/117)
72	*ifabp*	100% (15/15)	0% (0/0)	0% (0/0)	0% (0/63)	24% (15/63)	76% (48/63)	0% (0/54)	22% (12/54)	78% (42/54)
72	*ins*	100% (25/25)	0% (0/0)	0% (0/0)	10% (5/52)	12% (6/52)	79% (41/52)	0% (0/25)	12% (3/25)	88% (22/25)
72	*transf*	100% (27/27)	0% (0/0)	0% (0/0)	0% (0/115)	24% (28/115)	77% (87/115)	2% (2/100)	21% (21/100)	77% (77/100)

1 Hpf = hours post fertilization;

2 Gene expression was classified into one of three categories: none, weak wild type.

Summary of gene expression data reported in figures 3 and 4. DEAB treated embryos, as well as embryos from incrosses of *aldh1a2^um22^* heterozygotes and *aldh1a2^i26^* heterozygotes were assayed by in situ hybridization and their expression classified into one of three categories (no expression, wild type expression and weak expression). Embryos in each category are presented as a percent of the total number of embryos analyzed (actual numbers are given within parentheses).

### The Aldh Inhibitor DEAB Completely Blocks Expression of Endoderm Genes

We reasoned that the residual gene expression observed in *aldh1a2^um22^* and *aldh1a2^i26^* mutant embryos could either indicate that RA signaling is not completely required for expression of all genes in the endoderm, or it might indicate residual Aldh activity in the mutants. To test this further, we made use of DEAB (diethylaminobenzaldehyde), a competitive reversible inhibitor of all Aldh enzymes. DEAB has previously been reported to block development of fin buds and otic vesicles [29] and blocks expression of *hoxb1b*, *vhnf1*, *krx20* in rhombomere (r) 5, *val* in r5-6, *hoxd4a* and *efnb2a* in r7 of the hindbrain [42]. Zebrafish embryos treated with DEAB have been analyzed for a few endoderm markers [15], [22], [43], [44], [45]. In particular, *insulin::GFP* expression is lost in embryos treated with DEAB [15], [43], [44], [45]. Also, *foxa3* expression in the pancreas and liver and *vhnf1* expression in the pancreas is lost in DEAB treated embryos [45]. Loss of pharyngeal arches 3–5 was also seen when DEAB was used [46]. We find that treating zebrafish embryos with 10 uM DEAB starting at 8 hpf (see Figure S2 for DEAB titrations) blocks endoderm gene expression. Specifically, expression of *ins*, *pdx1*, *hhex*, *prox1*, *sid4*, *carbA*, *ifabp* and *transf* is completely lost in DEAB treated embryos (Figure 3B, F, J, N; Figure 4B, F, J, N, R; Table 2) while *sox17* expression is unaffected (not shown). Notably, treatment with lower concentrations (1–5 uM) of DEAB closely mimics the phenotypes observed in *aldh1a2^um22^* and *aldh1a2^i26^* mutant embryos (Figure S2). We conclude that Aldh activity is absolutely required for endoderm gene expression and that there is residual Aldh activity in *aldh1a2^um22^* and *aldh1a2^i26^* mutant embryos.

### Maternal *aldh1a2* Activity Persists in *aldh1a2^um22^* and *aldh1a2^i26^* Mutant Embryos

We next considered the likeliest source of residual Aldh activity in *aldh1a2^um22^* and *aldh1a2^i26^* mutant embryos. The expression patterns of *aldh1a3* (*raldh3*; observed primarily in developing eye, inner ear, pituitary gland and swim bladder) and *aldh8a1* (*raldh4*; found in liver and intestine, but not until day 2)[26], [27] make them unlikely candidates for providing Aldh activity in early pancreas development. In addition, *raldh1* is expressed in the dorsal retina and mesencephalic flexure in mice [26], but has not been found in zebrafish. Instead, we reasoned that there may be residual *aldh1a2* activity in the mutants. Since the *aldh1a2^um22^* and *aldh1a2^i26^* mutations are likely to be null mutations, we considered the most likely source of residual *aldh1a2* activity to be maternally deposited mRNA.

**Figure 5:**
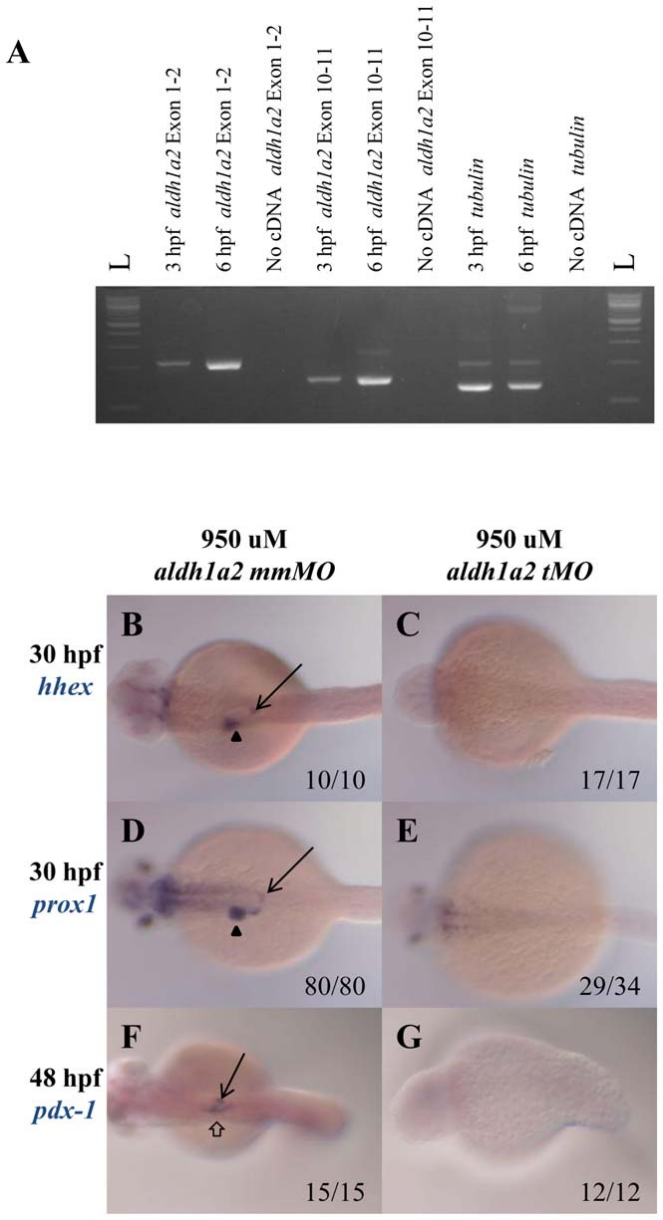
*aldh1a2* is maternally expressed and *aldh1a2* translational morpholino knocks down endoderm expression. A. PCR of 3 and 6 hpf wild type embryos using primers targeting exon1-2 and exon10-11 of *aldh1a2* reveals *aldh1a2* expression already at 3 hpf. A no DNA sample and amplification of tubulin is used as negative and positive controls. B–G. Wild type embryos were injected with either 950 uM *aldh1a2* mismatch (mm) morpholino (MO; B, D, F) or 950 uM of *aldh1a2* translational (tMO; C, E, G) and assayed for expression of *hhex* (B, C), *prox1* (D, E) or *pdx1* (F, G). Embryos are in dorsal view with anterior to the left.

To test this possibility, we first carried out RT-PCR on 3 hpf (before the onset of zygotic transcription) and 6 hpf (after the onset of zygotic transcription) zebrafish embryos. We find that *aldh1a2* mRNA is present already at 3 hpf (Figure 5A), consistent with a role for maternal *aldh1a2* mRNA. We reasoned that if the residual *aldh1a2* activity observed in the mutants is due to maternal mRNA, then blocking *aldh1a2* translation with antisense morpholino oligonucleotides (*aldh1a2* tMO) should produce the same phenotype as DEAB treatment. Indeed, we find that injecting *aldh1a2* tMO, completely blocks expression of *hhex* (Figure 5C), *prox1* (Figure 5E) and *pdx1* (Figure 5G), producing a phenotype indistinguishable from the DEAB phenotype and more severe than the *aldh1a2* mutant phenotype, while embryos injected with a mismatch MO control show wild type expression of all endoderm markers (Figure 5B, D, F). In contrast, we find that a MO targeting the *aldh1a2* exon 1/intron 1 splice junction (which should not affect already spliced maternal *aldh1a2* mRNAs) cannot fully block endoderm gene expression even at the highest concentration that could be tested (750 uM, not shown). We conclude that *aldh1a2* is the predominant *aldh* required for RA signaling during endoderm development and that *aldh1a2* has a significant maternal component.

## Discussion

We report results from an ENU (N-ethyl-N-nitrosourea) screen for genes involved in endocrine pancreas development. We characterize the *aldh1a2^um22^* allele, which corresponds to a glycine to arginine mutation in the catalytic domain of the Aldh1a2 protein. *aldh1a2^um22^* mutant embryos show similarities to embryos of two previously identified *aldh1a2* mutants, *neckless* (*nls* or *aldh1a2^i26/i26^*) and *no fin* (*nof*, *aldh1a2^u11/u11^*) [28], [34] in that all three mutants do not develop fin buds and have a swollen pericardium. We compare the endoderm phenotype of *aldh1a2^i26^* and *aldh1a2^um22^* mutant embryos to that of embryos treated with DEAB (a pan-Aldh inhibitor). Interestingly, endoderm markers are not uniformly lost in *aldh1a2* mutant embryos, but are lost in DEAB-treated embryos, suggesting residual Aldh activity in the mutants. We detect the presence of maternal *aldh1a2* transcripts and demonstrate that a morpholino targeting the *aldh1a2* translation start site copies the DEAB phenotype. We conclude that Aldh1a2 is the predominant Aldh enzyme acting in early pancreas development and that there is a significant role for maternally derived Aldh in this process.

### Aldh Activity Is Required for Pancreas Development

Disrupted RA signaling has broad effects such as shorter body length, curved body axis, lighter pigmentation, immobility, and a swollen pericardium. As a result, many developmental defects are observed, including neural crest cell death, the absence of limb buds and posterior branchial arches, small somites, and hindbrain segmentation defects, which have been known in general as VAD (vitamin A-deficiency syndrome) [47], [48], [49]. In mouse, a null mutation in the *Aldh1a2* gene mimics the hindbrain phenotypes associated with full VAD, establishing *Aldh1a2* as the main RA producing enzyme required in hindbrain development [29], [30], [50], [51]. As a result to losing RA, rhombomeric and gene expression boundaries posterior to rhombomere (r) 3 are lost [46], [47], [52], [53], [54], [55], [56]. In zebrafish embryos that are treated with DEAB to block Aldh activity, defects in anterior-posterior patterning of the neural tube also resemble severe VAD cases. The neural tube is strongly anteriorized and hindbrain development posterior to r4 is stopped. Also, loss of fin buds and reduction of branchial arches are observed [28], [34], [57]. This indicates a conserved role for Aldh enzymes in the production of RA required for hindbrain development in both zebrafish and mice.

RA is also involved in endoderm development in vertebrates. In mice, *Aldh1a2* is expressed in the dorsal pancreatic mesenchyme during pancreas specification and RA-responding cells reside in both pancreatic endoderm and mesenchyme [58]. As a result, defects in the endoderm are observed in the absence of RA. In particular, *Aldh1a2^−/−^* mice lack *Pdx1* and *Prox1* expression in the dorsal pancreatic bud but the ventral bud appears normal [2], [58]. Accordingly, *Insulin* and *Glucagon*-expressing cells do not develop and *Isl1* expression is severely decreased [58]. *Hlxb9*, expressed in the dorsal foregut endoderm, is also reduced [58]. Expression of *Foxa2* in the dorsoventral axis of the endoderm is not affected, indicating that early endoderm development is unaltered [2]. *Hhex* expression is not affected in the liver, suggesting that RA is not involved in liver development – similar to observations in *Xenopus* and avian embryos [2], [12], [13]. Treating *Xenopus* embryos with a RA receptor antagonist (BMS493) blocks dorsal pancreatic development, but does not affect ventral pancreatic development or the liver [12]. Similarly, in RA-deficient avian embryos or VAD (obtained from birds fed on a retinoid-deficient defined diet [59]), dorsal pancreas is lost but not ventral pancreas or liver [7], [48], [59]. Since *Xenopus* embryos treated with BMS493, VAD quail embryos and *Aldh1a2^−/−^* mutant mice display a similar phenotype - loss of dorsal pancreas but not ventral pancreas or liver – it appears that Aldh1a2 is the only Aldh acting in endoderm and that it is only necessary for dorsal pancreas development in these species. In contrast, blocking RA completely in zebrafish embryos eliminates all pancreas and liver gene expression. Embryos treated with DEAB lose *vhnf1* expression in the pancreas, *insulin*:GFP expression in the endocrine pancreas, *foxa3* expression in the pancreas and liver, and pharyngeal arches 3–5 are lost as well [15], [22], [43], [44], [45]. We treated embryos with 10 uM DEAB at 8 hpf and found that various endoderm markers expressed in the pancreas, liver, and intestine are lost, similar to embryos treated with BMS493 (pan-RAR antagonist) [15]. Also, injecting *aldh1a2* translational MO (tMO) knocks down *insulin* expression [8] and we find that *aldh1a2* tMO knocks down expression of genes such as *hhex* (liver and pancreas), *prox1* (liver and pancreas), and *pdx1* (pancreas and duodenum) as well (Figure 5).

Thus, there appears to be a conserved role for RA in pancreatic development among vertebrates, but mouse, *Xenopus* and avian embryos have restricted RA's role to the dorsal pancreas. The liver and ventral pancreas emerge adjacent to one another from the ventral endoderm in a default state as pancreas, but the liver receives signals from the cardiac mesoderm (FGF) to express liver markers [60]. Interestingly, the markers that continue to be expressed in *aldh1a2^um22^* and *aldh1a2^i26^* mutant zebrafish embryos are those expressed in the ventral pancreas and liver (*hhex*, *prox1*, *sid4*, *carbA*, and *transf*), indicating that less RA is needed to turn on expression of these genes, possibly consistent with an evolutionary phasing out of RA's involvement in these regions. Therefore, RA's role in ventral pancreas and liver development does not appear evolutionarily conserved among vertebrates. Other signaling factors may have taken precedence over RA in development of these regions in mouse. For instance, BMP and FGF signaling is necessary for liver development in mouse embryos, but inhibiting FGF and BMP signaling in zebrafish embryos leads to a decrease, not a loss, of *hhex* and *prox1* expression [61], [62], [63], [64].

Lastly, treatment with DEAB does not affect early endoderm gene expression in zebrafish embryos (*sox17*) or mutant mouse embryos (*FoxA2*) [2], indicating a conserved role that RA is not necessary for early endoderm development in vertebrates.

### The *aldh1a2^i26^*, *aldh1a2^u11^* and *aldh1a2^um22^* Alleles Likely Represent Null Mutations

The zebrafish *aldh1a2* mutant alleles exhibit defects in patterning of the neural tube and the endoderm, although the phenotype is not as severe as in DEAB-treated zebrafish embryos (Figs. 3, 4)[28], [34], *Aldh1a2^−/−^* mutant mice or VAD quail and rat embryos [28], [34]. Instead, it is similar to the phenotype we observe upon treatment with a low concentration of DEAB (Figure S2), as well as to a mild version of VAD seen in rat embryos and to partial rescue of *Aldh1a2^−/−^* mouse embryos by maternal application of RA [51], [55], [65]. Since Aldh activity appears absolutely required for pancreas formation (because DEAB-treated embryos lack endoderm gene expression, see above), the weaker phenotype of *aldh1a2* mutant zebrafish embryos could be explained if the *aldh1a2^i26^*, *aldh1a2^u11^* and *aldh1a2^um22^* alleles represent hypomorphic mutations that maintain some residual Aldh activity.

However, the mutations occurring in the *aldh1a2^i26^*, *aldh1a2^u11^* and *aldh1a2^um22^* alleles appear likely to be null mutations. In each case, the mutated residue is conserved across human, mouse, rat, *Xenopus*, and zebrafish [28], [34], indicating that amino acid sequence is important for the overall function of the Aldh1a2 protein and changing it will most likely affect the protein function. Furthermore, in each case, the mutation introduces a large charged residue (Gly -> Arg in *aldh1a2^i26^*, Thr -> Lys in *aldh1a2^u11^*, Gly -> Arg in *aldh1a2^um22^*). Such replacements are likely to affect the proper folding of the protein and therefore affect the catalytic function of Aldh1a2.

Further support for the idea that *aldh1a2^i26^*, *aldh1a2^um22^* and *aldh1a2^u11^* represent null mutations comes from rescue experiments, which indicate that the mutant proteins are not functional. When we injected *aldh1a2^um22^* embryos with mRNA containing the *aldh1a2^um22^* mutation, it could not rescue fin bud development (Figure 2 and Table 1). However, when we injected wild type *aldh1a2* mRNA, we were able to rescue fin bud development. The same was seen in rescue experiments using both *aldh1a2^i26^* and *aldh1a2^u11^* [28], [34]. Furthermore, overexpression of the *aldh1a2^um22^* mutant mRNA in zebrafish embryos did not affect development (not shown), further demonstrating that the *aldh1a2^um22^* allele is inactive. Together, this indicates that the *aldh1a2^i26^*, *aldh1a2^um22^* and *aldh1a2^u11^* mutations do not result in hypomorphic proteins, but represent null mutations.

### A Role for Maternal *aldh1a2* mRNA

If the *aldh1a2^i26^*, *aldh1a2^u11^* and *aldh1a2^um22^* alleles encode inactive Aldh1a2, the fact that *aldh1a2* mutant zebrafish embryos do not display a severe VAD phenotype suggest that Aldh activity must be coming from another source. The expression pattern of other *aldhs* rules them out as likely candidates and we therefore focused on maternal *aldh1a2* mRNA. We find that *aldh1a2* is expressed already at 3 hpf, albeit at somewhat lower levels – this lower level may explain the weak phenotype observed in the mutants. We also find that a MO targeting the exon 1/intron 1 splice site of *aldh1a2* (sMO, which should target only zygotic transcripts) produces a milder phenotype (not shown) and that lower doses of *aldh1a2* tMO (500 uM) permit some expression of *pdx1*, similar to the phenotype observed in *aldh1a2^um22^* and *aldh1a2^i26^* mutant embryos). We also note that after treating with DEAB, it became clear that *aldh1a2^i26^* embryos still have some RA activity since DEAB treated embryos display a severe phenotype similar to VAD [34], [57], [65]. This residual *aldh1a2* is most likely due to maternally supplied mRNA.

In contrast, Aldh enzymes do not appear to be deposited maternally in other vertebrates. In particular, the fact that *aldh1a2* mutations in mice mimic the VAD phenotype [47], [48], [49], suggests that there are no maternally contributed Aldhs in the mouse. In *Xenopus*, both retinol and retinaldehyde are present in embryos before gastrulation, indicating that RDHs may be present (possibly maternally deposited) [57], [66]. Furthermore, microinjection of *Aldh1* or *Aldh1a* induces premature RA signaling in *Xenopus* [67] by acting on this retinaldehyde pool, suggesting that maternally deposited Aldhs are not present in the *Xenopus* embryo [28], [50], [67], [68]. It is not clear why *aldh1a2* is maternally deposited in zebrafish, but our observation that treatment with DEAB before gastrulation results in death or severely deformed embryos (not shown) suggests that there may be an early role for *aldh1a2* in zebrafish embryos.

## Supporting Information

Figure S1 Double in situ in *aldh1a2^um22^* and *aldh1a2^i26^* mutant embryos. Wild type (A, F), *aldh1a2^um22^* (B, C, G, H) and *aldh1a2^i26^* (D, E, I, J) embryos were assayed for expression of *prox1/ins* at 30 hpf (A-E) and *carbA/ins* at 72 hpf (F-J). *Ins* expression is detected in purple, while *prox1* (A-E) and *carbA* (F-J) are detected in red. We do not observe any correlation in the extent of residual expression by these genes in individual embryos.
(3.80 MB TIF)

Figure S2 Titration of DEAB and *aldh1a* tMO. Wild type (A), *aldh1a2^um22^* mutant (B), *aldh1a2^i26^* mutant (C), DEAB-treated (D-G) and *aldh1a2* tMO-injected (H-K) embryos were assayed for *pdx1* expression at 48 hpf. DEAB and *aldh1a2* tMO was titrated as indicated (D-G and H-K, respectively). Black arrows indicate pancreas expression and open arrows indicate duodenum expression of *pdx1*. Note that intermediate concentrations of DEAB (1 uM, panel E) and *aldh1a2* tMO (250–500 uM, panels I, J) produce similar phenotypes to the *aldh1a2^um22^* and *aldh1a2^i26^* mutants. Embryos are in dorsal view with anterior to the left.
(6.16 MB TIF)
