# Supplementary figures and images for: Maternal and Zygotic aldh1a2 Activity Is Required for Pancreas Development in Zebrafish
Source: PLoS One. 2009 Dec 11;4(12):e8261. doi: 10.1371/journal.pone.0008261 (PMC2788244; doi:10.1371/journal.pone.0008261)

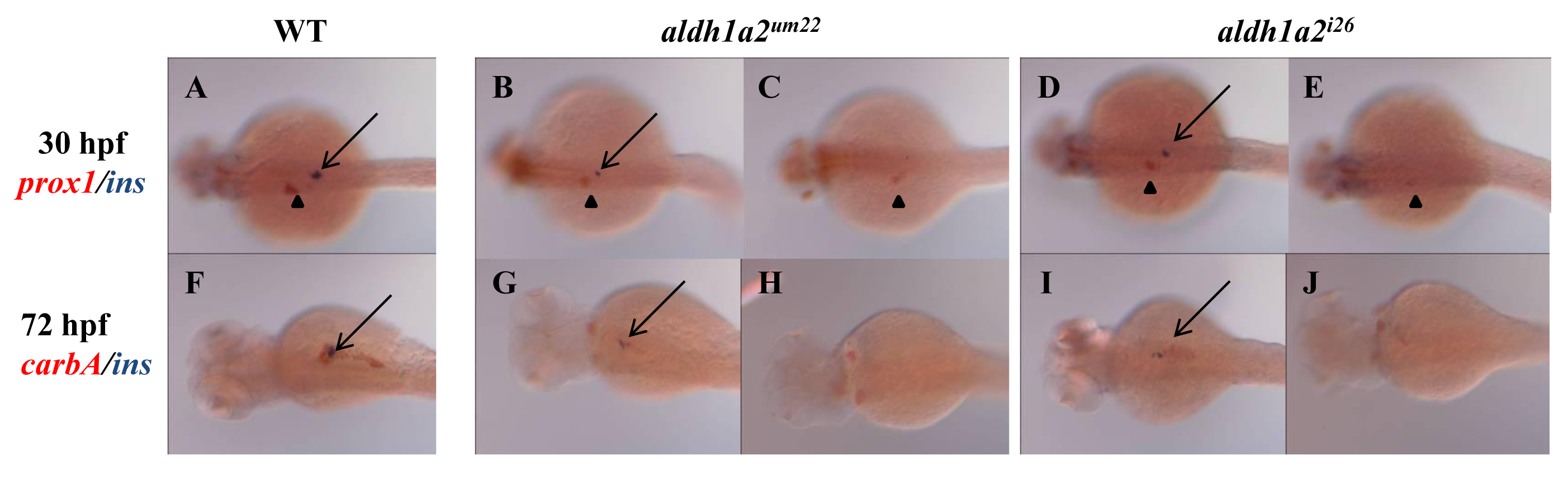

Supplement: Figure S1 — Double in situ in aldh1a2um22 and aldh1a2i26 mutant embryos. Wild type (A, F), aldh1a2um22 (B, C, G, H) and aldh1a2i26 (D, E, I, J) embryos were assayed for expression of prox1/ins at 30 hpf (A-E) and carbA/ins at 72 hpf (F-J). Ins expression is detected in purple, while prox1 (A-E) and carbA (F-J) are detected in red. We do not observe any correlation in the extent of residual expression by these genes in individual embryos. (3.80 MB TIF) [file pone.0008261.s001.tif]

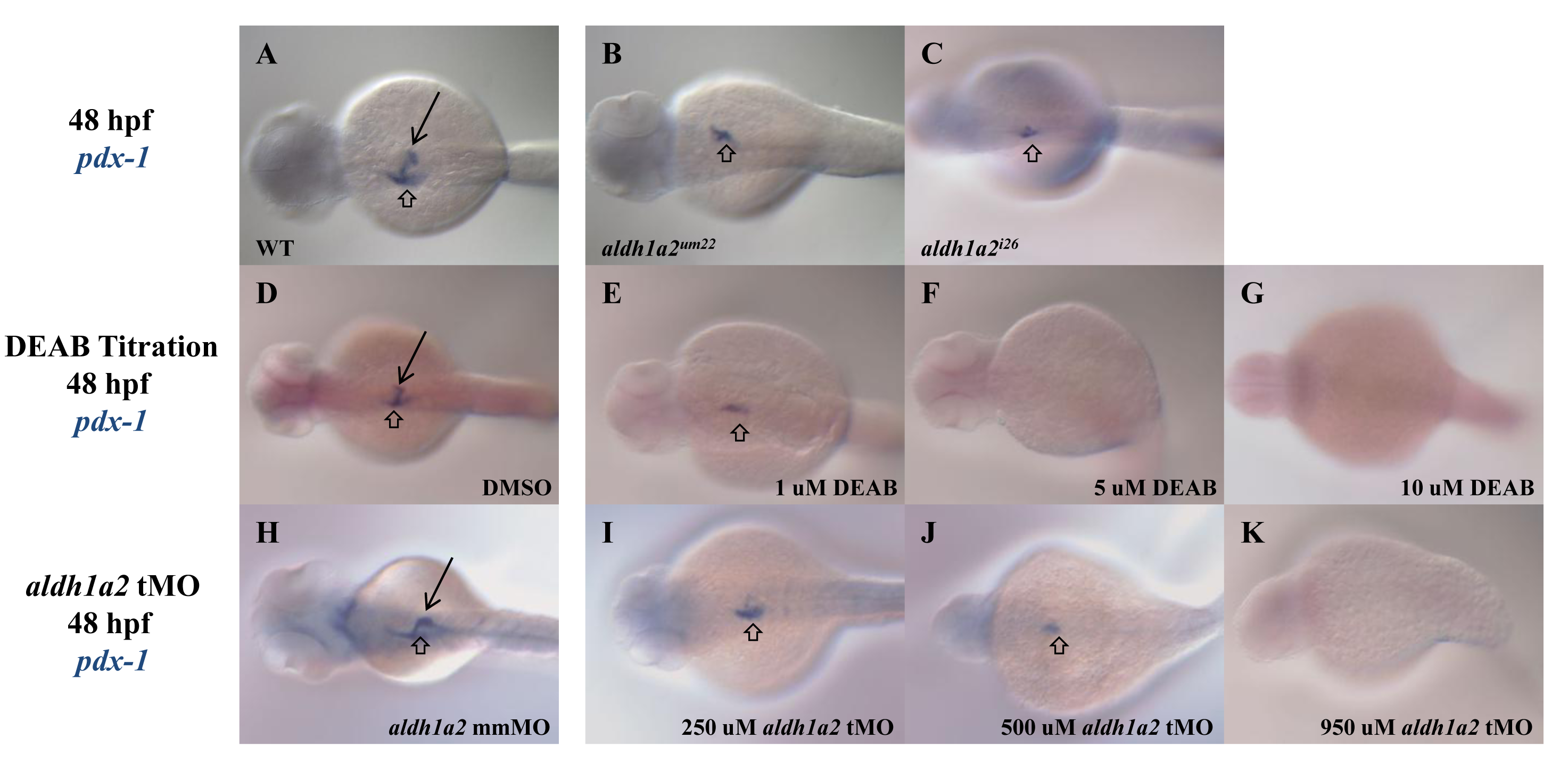

Supplement: Figure S2 — Titration of DEAB and aldh1a tMO. Wild type (A), aldh1a2um22 mutant (B), aldh1a2i26 mutant (C), DEAB-treated (D-G) and aldh1a2 tMO-injected (H-K) embryos were assayed for pdx1 expression at 48 hpf. DEAB and aldh1a2 tMO was titrated as indicated (D-G and H-K, respectively). Black arrows indicate pancreas expression and open arrows indicate duodenum expression of pdx1. Note that intermediate concentrations of DEAB (1 uM, panel E) and aldh1a2 tMO (250–500 uM, panels I, J) produce similar phenotypes to the aldh1a2um22 and aldh1a2i26 mutants. Embryos are in dorsal view with anterior to the left. (6.16 MB TIF) [file pone.0008261.s002.tif]
